# Key role of microbial necromass and iron minerals in retaining micronutrients and facilitating biological nitrogen fixation in paddy soils

**DOI:** 10.1016/j.fmre.2024.02.007

**Published:** 2024-03-06

**Authors:** Li-Xin Xu, Fei Wang, Yao Yao, Minjie Yao, Yakov Kuzyakov, Guang-Hui Yu, Cong-Qiang Liu

**Affiliations:** a Institute of Surface-Earth System Science, School of Earth System Science, Tianjin Key Laboratory of Earth Critical Zone Science and Sustainable Development in Bohai Rim, Tianjin University, Tianjin 300072, China; b Institute of Soil and Fertilizer, Fujian Academy of Agricultural Sciences, Fuzhou 350013, China; c Engineering Research Center of Soil Remediation of Fujian Province University, College of Resources and Environment, Fujian Agriculture and Forestry University, Fuzhou 350002, China; d Department of Soil Science of Temperate Ecosystems, Department of Agricultural Soil Science, University of Gӧttingen, Gӧttingen 37073, Germany; e Agro-Technological Institute, Peoples Friendship University of Russia (RUDN University), Moscow 117198, Russia

**Keywords:** Amino sugars, Functional genes, Long-term fertilization, Metagenomics, Synchrotron radiation based spectromicroscopy, Trace metals

## Abstract

Paddy fields, as the largest anthropogenic wetlands on Earth, face a high risk of micronutrient loss through surface runoff and leaching due to their frequent irrigation-drainage cycles, as well as removal with crop harvest. While micronutrient’s losses largely impede biological nitrogen fixation (BNF) in soils, agricultural practices and mechanisms that retain micronutrients and thus increase BNF in paddy soils remain underexplored. Here we showed that the long-term (40 years) application of fertilizers increased the contents of microbial necromass in paddy soils by 20%–43% compared to the unfertilized control. In particular, long-term organic fertilizations increased poorly crystalline minerals through intensifying mineral weathering, which further contributed to the increased stable carbon burials in paddy soils. Synchrotron radiation based spectromicroscopy analysis provided direct evidence demonstrating a differential control of hydroxyl functional groups from mineral surfaces on C functional groups at the submicron scale in paddy soils. Notably, microbial necromass and short-range ordered minerals had a strong correlation with the content and bioavailability of micronutrients in paddy soils, indicating their key roles in the retention of micronutrients. Metagenomic sequencing analysis further indicated that the content and bioavailability of micronutrients were strongly correlated with the abundance of the key N-fixing genera (i.e., Azospirillum and Bradyrhizobium). Unexpectedly, structural equation modeling (SEM) identified that microbial necromass exerted the strongest control on N-fixing genera, highlighting an underappreciated role of microbial necromass as a reservoir of micronutrients. Based on micronutrient's bioavailability and metagenomic sequencing, we conclude that micronutrients are the key factor for BNF in paddy soils, offering significant implications for managing BNF in paddy soils.

## Highlights

• Manure and straw inputs substantially increased the micronutrients in paddy soils.
• Mo and V had a strong correlation with microbial necromass and reactive minerals.
• Micronutrients were strongly correlated with the abundance of key N-fixing genera.
• Microbial necromass exerted the strongest control on N-fixing genera.

## Graphical abstract

**Figure:**
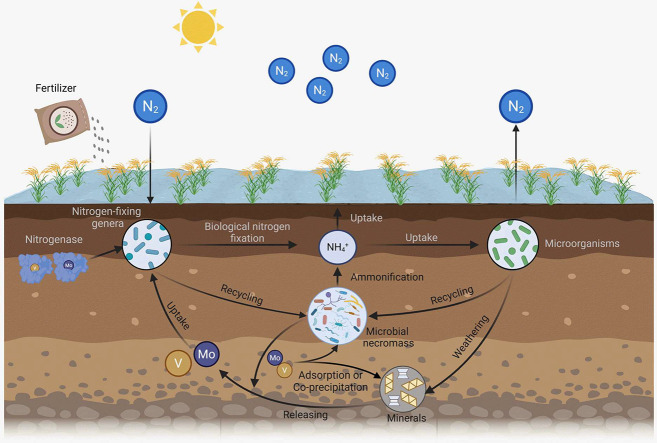

## 1 Introduction

Paddy fields constitute the largest anthropogenic wetlands on Earth, accounting for approximately 12% of global cropland area [1,2]. Because of their irrigation-drainage cycles [3,4], paddy fields are at high risk of micronutrient loss, e.g., molybdenum (Mo) and vanadium (V), through surface runoff, leaching, and erosion, as well as removal by harvest-larger as with any other crops as rice will be cropped 2–3 times per year. Essentially, these micronutrients are the cofactors of the isoform of the nitrogen (N) fixing nitrogenase (e.g., Mo-nitrogenase and V-nitrogenase) [5,6]. Therefore, the loss of micronutrients in paddy soils is expected to decrease biological N fixation (BNF) that converts un-reactive N_2_ into bioavailable ammonia (NH_3_ or NH_4_^+^) by N-fixing bacteria in soils [7,8].

Indeed, as the dominant biological route for bioavailable N inputs into Earth’s ecosystems [8], BNF by symbiotic bacteria in roots of higher plants, by associative bacteria living in the rhizosphere of plants, and asymbiotic bacteria in terrestrial ecosystems is approximately 40–110 teragrams (Tg) N/year [8,9]. Current viewpoints indicate that BNF is primarily affected by factors such as phosphorus depletion (*P limitation hypothesis*), N richness (*N richness hypothesis*), and an imbalance of substrate stoichiometry (*stoichiometry hypothesis*) [10,11]. However, the significance of micronutrients in the BNF process within agricultural fields has been largely neglected. Given the distinct cultivation practices in paddy soils, our study specifically emphasizes the importance of increasing the storage of micronutrients and their bioavailability to N-fixing microorganisms is the key to increase BNF while simultaneously reducing the applications of synthetic N fertilizers in paddy soils without sacrificing food production.

The storage and bioavailability of micronutrients in soils are controlled by soil organic matter (SOM) and nano-sized mineral phases [12]. Micronutrients can bind to SOM through specific adsorption and ligand exchange, which prevents their leaching and surface runoff [12,13]. Microbial necromass, the remains of dead microorganisms, comprises third to half of soil organic carbon (SOC) in topsoil across various ecosystems [14]. Microbial necromass provides abundant charged organic molecules [15] and thus may serve as a reservoir for essential micronutrients. Notwithstanding, the roles of microbial necromass in the retention of micronutrients in soils remain underexplored, and for paddy soils is not known.

Besides microbial necromass acting as “microbial C pump” [16], minerals can also adsorb microbial necromass or other SOM components to form “mineral C pump” [17]. Mineral associated organic matter (MAOM) can contribute to C persistence for extended timescales, spanning centuries to millennia [18]. Formation of strong organic complexes with minerals not only stops micronutrient leaching [6,19] but also acts as a sustainable reservoir to impede their rapid uptake by N-fixing bacteria [5]. Among mineral matrix, poorly crystalline minerals or reactive minerals, extracted by acid oxalate [20] and citrate-bicarbonate-dithionite (CBD) [21], respectively, are key to retain micronutrients [19]. These minerals are mainly composed of short-range ordered (SRO) minerals with large surface area and reactive sites [22]. Understanding the importance of SOM and minerals in controlling micronutrient behaviors is key to determine the bioavailability of micronutrients to N-fixing genera and the N supply capacity of paddy soils. To date, the linkage between microbial necromass, minerals and micronutrients has not been built, impeding our understanding on BNF in paddy soils.

Here, the objectives of this study were to (1) examine how long-term fertilization affects the storage and bioavailability of micronutrients in paddy soils; (2) identify edaphic factors controlling the storage and bioavailability of micronutrients; and (3) explore the N-fixation genera responsible to the availability of micronutrients. We hypothesized that (1) the storage of micronutrients in paddy soils is jointly controlled by microbial necromass and poorly crystalline minerals, offering ample binding sites; and (2) the bioavailability of micronutrients primarily relies on the recycling of microbial necromass via mineralization and decomposition, alongside the essential role of reduction or/and acidification of poorly crystalline minerals, to mobilize micronutrients. To test our hypotheses, a well-controlled long-term (40 years) field experiment of different fertilization on paddy soils in the subtropical region, China, was used. Together, our findings provide compelling evidence that microbial necromass and iron oxides exerted the strong control on N-fixing genera, highlighting an underappreciated role of microbial necromass and iron oxides as a reservoir of micronutrients that drives the variation in N-fixing genera and BNF in soils.

## 2 Materials and methods

### 2.1 Site description and soil sampling

The long-term fertilization experiment was established in 1983 in Minhou County (119°04′10″ *E*, 26°13′31″ *N*), Fujian Province, China, in an area with a subtropical monsoon humid climate. The mean annual temperature is 19.5 ℃, mean annual precipitation is 1,350 mm, and mean annual sunshine hours are 1,810 h. The soil is yellow muddy soil, as classified by the Chinese Soil Taxonomy, or Anthrosol, according to the Food and Agriculture Organization (FAO) of the United Nations classification. Four fertilizations in this field were selected [23], including no fertilization (control), mineral N, phosphorus and potassium (NPK) fertilization, NPK combined with cow manure (NPKM) fertilization, and NPK combined with straw (NPKS) fertilization. Each treatment was replicated three times, with a plot area of 12 m^2^ (Fig. S1). The fertilization rates and nutrient contents of the applied cattle manure and straw are provided in Tables S1 and S2. The management practices were consistent across all treatments, except for fertilizer application.

In September 2022, composite topsoil (0–20 cm) samples were collected from multiple points along the diagonal line of experimental plots using a 5 cm diameter auger. The samples were mixed, and then divided into two splits for further analysis. One split was air-dried and sieved for analyses of micronutrients, amino sugars, reactive minerals, and other physico-chemical properties, while the other was stored in −80 °C refrigerators for molecular analyses, including metagenomic sequencing and high-throughput quantitative PCR (HT-qPCR) [24].

### 2.2 Amino sugar determination

Amino sugars were used as an indicator of microbial necromass in the soil [25] and the details can be found in the SI. In total, three amino sugars, including glucosamine (GluN), galactosamine (GalN), and muramic acid (MurN), were quantified by Dionex RS 3400 fluorescence detector (HPLC, Dionex Ultimate 3000, Thermo Fisher Scientific, USA). GluN was originated from both fungal cell walls (mainly chitin) and bacterial cell walls (mainly peptidoglycan), while MurA was derived from bacterial cell walls [14]. The origin of GalN has not been identified [26]. Accordingly, the bacterial necromass C (B-necromass C) and fungal necromass C (F-necromass C) were calculated as follows (Eqs. (1) and (2)).

(1) B−necromassC=MurA×45

(2) F−necromassC=(GluN/179.17−2×MurA/251.23)×179.17×9

where 45 and 9 are conversion factors; 179.17 and 251.23 are the molecular weights of GluN and MurA, respectively. Total necromass (T-necromass) was calculated as the sum of B-necromass and F-necromass. Moreover, T-necromass was further divided by SOC to denote their contributions to C storage. Other details are provided in the Supporting Information.

### 2.3 Analyses of reactive minerals and mineral-associated carbon

Reactive Fe minerals (Fe_CBD_) were extracted using a 0.1 M citrate bicarbonate dithionite (CBD) solution [21]. Specifically, 0.5 g of soil was shaking with 30 mL of 0.68 M sodium citrate solution and 0.4 g of sodium dithionite for 14 h. As control experiments, soils were extracted with NaCl instead of CBD. Then, the suspensions were filtered by 0.45-µm filters and quantified by ICP-OES. Fe-bound organic C (OC, %) was determined using Eq. (3):

(3) $$\text{Fe}-\text{bound}\;\text{OC}\;\left(\%\right)\;=\;\left(OC_{\text{NaCl}}-OC_{\text{CBD}}\right)/\text{SOC}\;\times \;100$$

where OC_NaCl_ and OC_CBD_ denote the OC content of NaCl- and CBD-treated soil residues, respectively. Although CBD extraction may not completely reduce reactive Fe (Fe_CBD_), resulting in less efficient measurement of Fe-bound OC [27], it is still a widely used method to quantify OC bound to reactive Fe minerals [21].

Poorly crystalline Fe and Al (oxyhydr)oxides (Fe_o_ and Al_o_) were extracted with 0.275 M acid ammonium oxalate at pH 3.25, with a soil solution ratio of 1:100. Ammonium oxalate selectively removes SRO minerals such as ferrihydrite and allophane, but is a poor extractant of layer silicates and does not extract crystalline minerals [28]. The mixture was shaken in the dark for 4 h, followed by the calculation of SRO minerals (SRO = Al_o_ + 1/2Fe_o_) [29].

### 2.4 Synchrotron radiation based infrared spectromicroscopy

Paddy soil samples were first placed on a glass fibre filter paper and humidified over a period of 24 h [30]. Subsequently, soil particles were manually selected and frozen in cryomicrotome (Cyrotome E, Thermo Shandon Limited, UK) at −20 °C. Finally, thin sections (1 µm in thickness) of the soil particles were obtained, using a stainless steel knife without the need for any embedding medium [31]. These thin sections were then transferred to MirrIR Low-E microscope slides (Kevley Technologies, Ohio, USA), which possess infrared-reflecting properties, facilitating the SR-FTIR measurements.

These thin sections were analyzed in triplicate using the Thermo Nicolet 6700 FTIR spectrometer and a continuum infrared microscope. The scanning wavenumber range was set from 4,000 to 650 cm^−1^, and the measurements were conducted in reflection mode at the BL01B Beamline of the Shanghai Synchrotron Radiation Facility (SSRF). The infrared microscope’s key detection parameters were configured as follows: aperture size of 10 µm × 10 µm, step size of 10 µm × 10 µm, resolution of 4 cm^−1^, and 64 scans [31].

Following spectral map calibration with OMNIC 9.0 (Thermo Fisher Scientific Inc.), the characteristic peak intensity and spatial distribution position of each functional group were accurately depicted. The specific IR absorbances targeted for analysis were as follows: 3,621 cm^−1^ (mineral-OH), 2,922 cm^−1^ (lipids), 1,643 cm^−1^ (amides), and 1,030 cm^−1^ (polysaccharides) [32]. By utilizing the intensities of absorption peaks corresponding to each functional group, a false-color 2D map was generated for image processing purposes. These intensities, along with linear regression, were employed to evaluate the spatial correlation between mineral-OH (hydroxyl functional groups of mineral surfaces) and selected C functional groups.

### 2.5 Micronutrient determination

To determine the total contents of Mo (Mo_t_) and V (V_t_) in soils, a mixture of HNO_3_-HCl-HF acid with the ratio of 6:3:2 was used to digest the soil in a microwave digestion system (CEM Corp., Matthews, N.C., USA) [19]. The bioavailability of Mo (Mo_a_) and V (V_a_) was determined using a modified ammonium oxalate extraction method [19]. Approximately 5 g of soil was extracted with 1.0 M ammonium oxalate solution (pH = 3.3, solid-liquid ratio of 1:10 w/v). After shaking for 30 min at 300 rpm and room temperature on a reciprocal shaker [12], the suspension was filtered. The total contents and bioavailability of Mo and V were analyzed by Inductively Coupled Plasma Mass Spectrometry (7900 ICP-MS, Agilent Technologies, USA).

### 2.6 Nitrogen fixation genera and functional gene analyses

Soil deoxyribonucleic acid (DNA) was extracted by the PowerMax Soil DNA isolation kit (MOBIO Laboratories, CA, USA), after which the DNA samples were tested for total amount and purity. The metagenomic sequencing was commissioned by Meige Gene Technology Co., Ltd. After obtaining the metagenomic sequencing data for each sample, quality control was performed using Trimmomatic to remove the low-quality data and obtain the Clean Reads. The de novo splicing of the Clean Reads was performed using MEGAHIT. Open reading frame prediction was achieved using MetaGeneMark [33]. To obtain the annotation information of the related N cycle functional genes [34], this database integrates the information of N cycle functional genes in KEGG, COG, eggNOG and Uniprot. A total of 68 genes from N cycling functional gene families were included.

In total, 23 genes including 22 functional genes related to N cycling and one 16S rRNA gene (Table S3) were assessed using Quantitative microbial element cycling (QMEC), a HT-qPCR-based chip, to evaluate the microbial functional potential [35]. The amplification process was carried out using the SmartChip Real-time PCR system (Wafergen, Fremont, CA) in a 100 nL reaction system. Each primer set was subjected to triplicate qPCR reactions, and a non-template negative control was included in each run. Relative gene abundance was determined as the ratio of the abundances of functional genes to the 16S rRNA gene. Absolute gene abundance was determined based on the absolute 16S rRNA gene copy number using conventional qPCR [35]. Sequence data are deposited in the NCBI Sequence Read Archive (SRA) database (PRJNA1000419).

### 2.7 Statistical analysis

All statistical analyses were performed using the IBM SPSS Statistics 26, while figures were plotted using Origin 2022 and *R* software. Analysis of significant differences was performed using Duncan post-hoc multiple comparisons in the one-way Analysis of variance (ANOVA) test, and *p* = 0.05 was used as the significance level of error probability. Letters on the graphs indicate significant differences [19]. Pair correlations between micronutrients and soil edaphic factors were performed using Pearson's correlation analysis [36]. A structural equation modeling (SEM) was constructed by using the AMOS software to analyze the direct and indirect effects of edaphic factors on N fixing genera, respectively. All data in the figures and tables are the means ± SE.

## 3 Results

### 3.1 Microbial necromass and minerals in long-term fertilized paddy soils

**Fig. 1:**
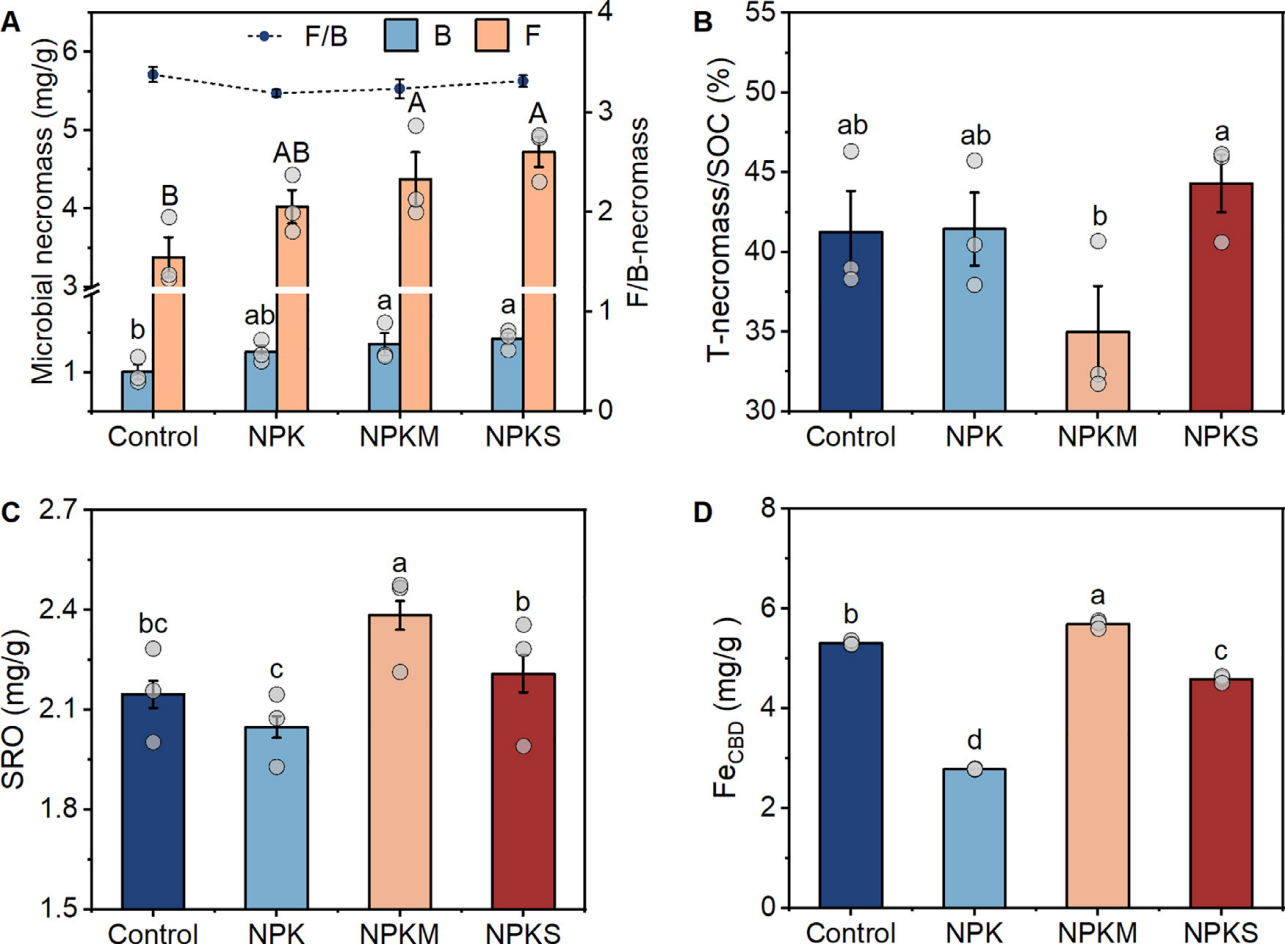
**Microbial necromass and poorly crystalline minerals in long-term fertilized paddy soils.** (A) Microbial necromass; (B) T-necromass/SOC; (C) SRO; (D) Fe_CBD_. B, bacterial necromass; F, fungal necromass; F/B-necromass, fungal/bacterial necromass; T-necromass/SOC, total necromass C/ soil organic carbon. SRO (short-range-ordered) minerals are poorly crystalline Fe and Al (oxyhydr)oxides (Fe_o_, Al_o_), which are extracted using acid ammonium oxalate. Fe_CBD_ refers to the reactive Fe minerals extracted using citrate bicarbonate dithionite (CBD) method. Control, no fertilizers; NPK, mineral fertilizers; NPKM, mineral fertilizer plus cow manure; NPKS, mineral fertilizer plus straw. Significant differences between fertilizations were determined using one-way ANOVA followed by Duncan’s multiple test at *p* < 0.05. Different letters positioned above the histogram signify statistically significant differences, with uppercase and lowercase letters denoting fungal and bacterial necromass, respectively. Data are means ± SE (*n* = 3).

To identify the edaphic factors affecting the content and bioavailability of micronutrients in paddy soils, microbial necromass and poorly crystalline minerals were further characterized (Fig. 1). The long-term application of fertilizers raised the contents of microbial necromass in paddy soils by 20%−43% compared to the unfertilized control (Fig. 1A). Specifically, the bacterial and fungal necromass C contents increased by 20%−26% for NPK, 30%−36% for NPKM, and 40%−43% for NPKS, respectively. Conversely, the fungal/bacterial necromass C ratio remained stable at around 3.2–3.4 across all the fertilizations (Fig. 1A), indicating that fungal necromass was more dominant relative to bacterial, and the application of fertilizers did not alter the ratio of fungal and bacterial necromass. These fungal and bacterial necromass harbored numerous binding sites capable of adsorbing positively charged minerals, thereby playing a role in the adsorption and ligand exchange of negatively charged micronutrients (MoO_4_^2−^ and VO_3_^−^).

Microbial necromass contributed to approximately 35%−43% of SOC (Fig. 1B). While the soil with NPKM had a higher total necromass C content compared to NPK and Control (Figs. 1A and S2A), the ratio of total microbial necromass in SOC in the NPKM was the lowest (Fig. 1B) because of its high SOC content following long-term manure application (Table S4). Contrary to the trend of total necromass C/SOC ratio (Fig. 1B), Fe-bound OC was the highest in the soil with NPKM (Fig. S2B). The application of fertilizers increased the Fe-bound OC under NPKM for 145%, under NPKS for 94%, and for 42% under NPK compared to the unfertilized soil (Fig. S2B). As a result, the high SOC proportion in the NPKM was protected by reactive minerals. Taken together, the long-term organic inputs substantially increased the stable C pool, including microbial necromass and Fe-bound OC, in paddy soils.

The Fe_t_/Fe_CBD_ in the soil (Table S4) serves as an indicator for evaluating the extent of soil weathering. This decrease signified the gradual release of extractable iron from minerals, providing confirmation of intensified mineral weathering. Of all the examined fertilizations, NPKM had the highest contents of SRO and Fe_CBD_, while NPK had the lowest (Fig. 1C, D). Specifically, NPKM showed an increase of approximately 16% for SRO and 105% for Fe_CBD_ compared to the NPK (Fig. 1C, D), revealing an increased formation of poorly crystalline minerals by organic fertilizations. In addition, dissolved Fe had a similar trend with SRO in the long-term fertilized paddy soils (Fig. S3), possibly attributable to the intensified mineral weathering. Therefore, long-term organic fertilizations, in particular manure inputs, increased poorly crystalline minerals (mainly SRO) through intensifying mineral weathering, which further contributed to the increased stable C pool in paddy soils.

### 3.2 Control of mineral OH groups on carbon functional groups in paddy soils

**Fig. 2:**
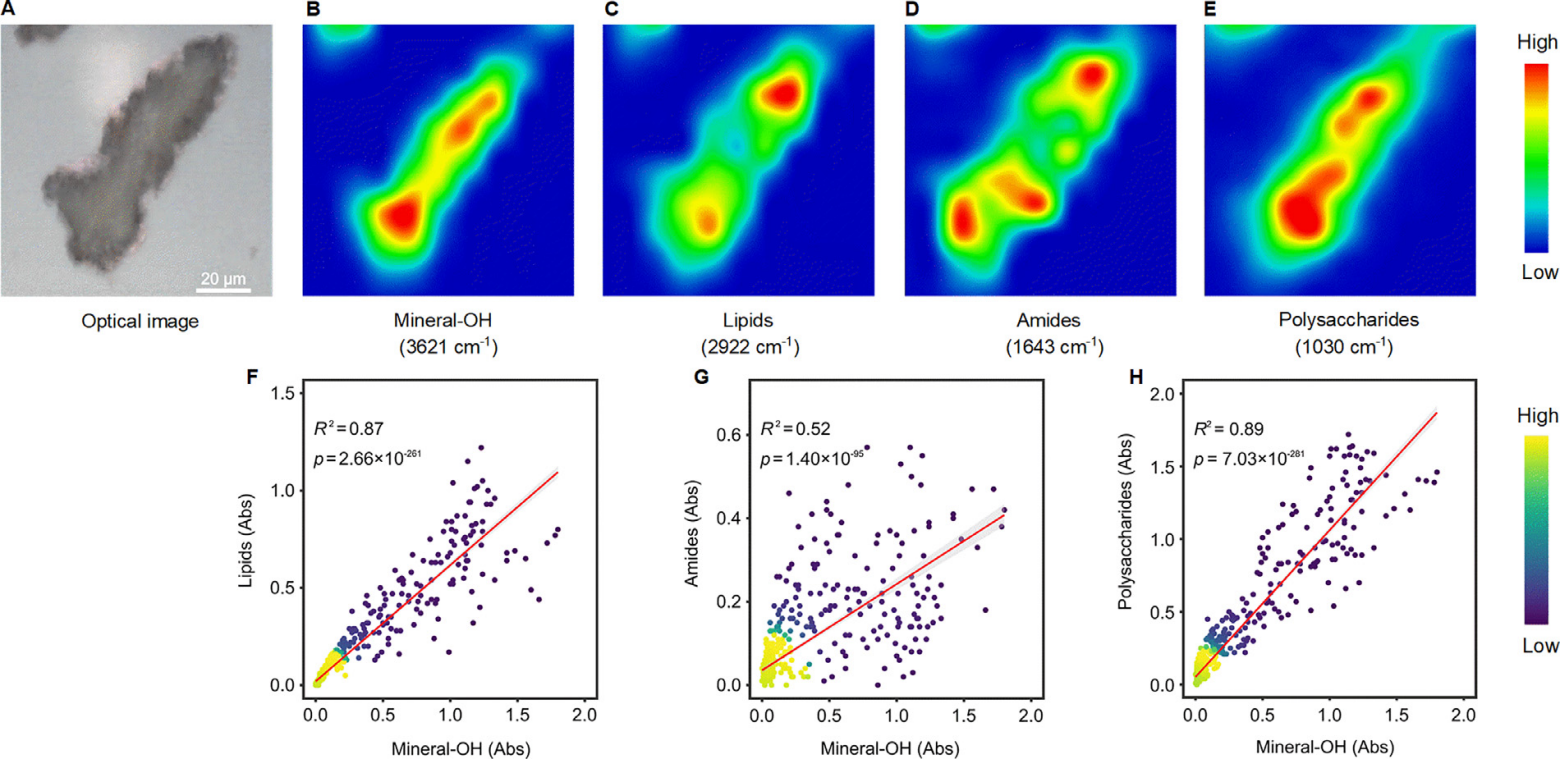
**Mapping and correlations of mineral and carbon functional groups in long-term fertilized paddy soils.** (A) Typical optical image. (B) Mapping of mineral OH (3,621 cm^−1^). (C) Mapping of lipids (2,922 cm^−1^). (D) Mapping of amides (1,643 cm^−1^). (E) Mapping of polysaccharides (1,030 cm^−1^). (F) Correlation between mineral OH and lipids. (G) Correlation between mineral OH and amides. (H) Correlation between mineral OH and polysaccharides. Mineral OH, hydroxyl functional groups of mineral surfaces. The colour bar is a relative scale for each peak height in (B-E) and does not allow quantitative comparison between peaks. In contrast, in (F-H), it signifies density based on the numbers of data points in (B-E). *N* = 587 in (F-H).

To visualize the spatial correlations between mineral and organic functional groups in paddy soils at the micron scale, synchrotron radiation based- Fourier transform infrared microspectroscopy (SR-FTIR) analysis was employed (Fig. 2). Overall, hydroxyl functional groups of mineral surfaces and major C functional groups showed a heterogeneous but different distribution patterns in long-term fertilized paddy soil particles (Figs. 2A-E and S4). Notably, across all examined samples, mineral-OH possessed a better correlation with lipids and polysaccharides than amides, regardless of fertilization treatments (Fig. 2F-H; Table S5). This suggests that lipids and polysaccharides had a more tightly bound to mineral microsites than amides at the submicron scale, revealing a preferential preservation of lipids and polysaccharides by minerals in paddy soils. Collectively, the spectromicroscopy analysis showed compelling evidence for a differential control of hydroxyl functional groups from mineral surfaces on C functional groups at the submicron scale in paddy soils, revealing a selective protection of minerals on soil C burial at the functional group level.

### 3.3 Micronutrients in long-term fertilized paddy soils

**Fig. 3:**
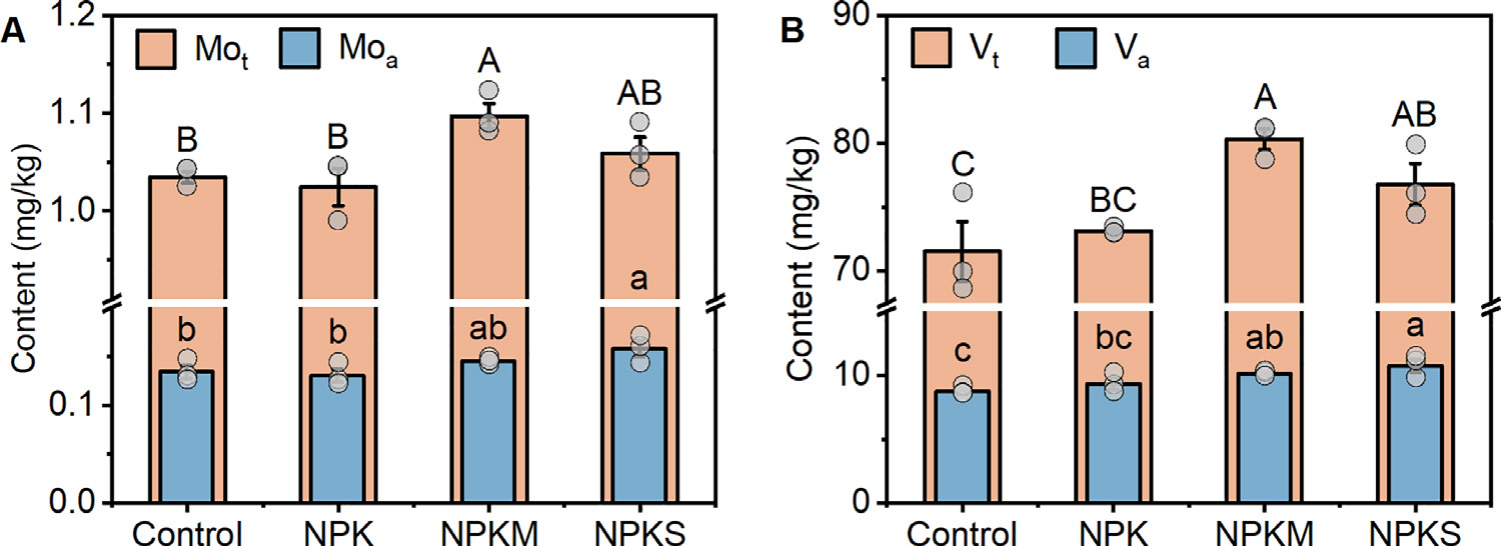
**Micronutrients in long-term fertilized paddy soils.** (A) Mo_t_; (B) V_t_; (C) Mo_a_; (D) V_a_. Mo_t_, total Mo; V_t_, total V; Mo_a_, bioavailable Mo; V_a_, bioavailable V. Control, no fertilizers; NPK, mineral fertilizers; NPKM, mineral fertilizer plus cow manure; NPKS, mineral fertilizer plus straw. Significant differences between fertilizations were determined using one-way ANOVA followed by Duncan’s multiple test at *p* < 0.05. Different letters positioned above the histogram signify statistically significant differences, with uppercase and lowercase letters denoting the total and bioavailable micronutrients, respectively. Data are means ± SE (*n* = 3).

After 40 years of fertilization, both NPKM (6%-12%) and NPKS (3%-7%) increased the contents of total Mo and V in paddy soils compared to the unfertilized control, while NPK only slightly (−1%-2%) changed them (Fig. 3A, 3B). Similar to the total contents of micronutrients, NPKS and NPKM had a higher levels of bioavailable Mo and V than the control and NPK (Fig. 3C, 3D). Specifically, compared to the control and NPK, bioavailable Mo and V increased for 17%-23% under NPKS, while only for 8%-16% under NPKM. Across all the examined fertilization types in paddy soils, the content and bioavailability of total V was over 61 and 49 times higher than those of Mo (Fig. 3A, 3B), respectively. By connecting the total content and bioavailable Mo and V with grain production (Fig. S5), we found that the increased micronutrients (except for bioavailable Mo) closely correlated with the production of rice grains (Fig. S6), suggesting the contribution of Mo and V to food production. Taken together, these results indicated that organic fertilization may offer a feasible pathway to increase micronutrients that may contribute to food production.

### 3.4 Control of microbial necromass and minerals on micronutrients

**Fig. 4:**
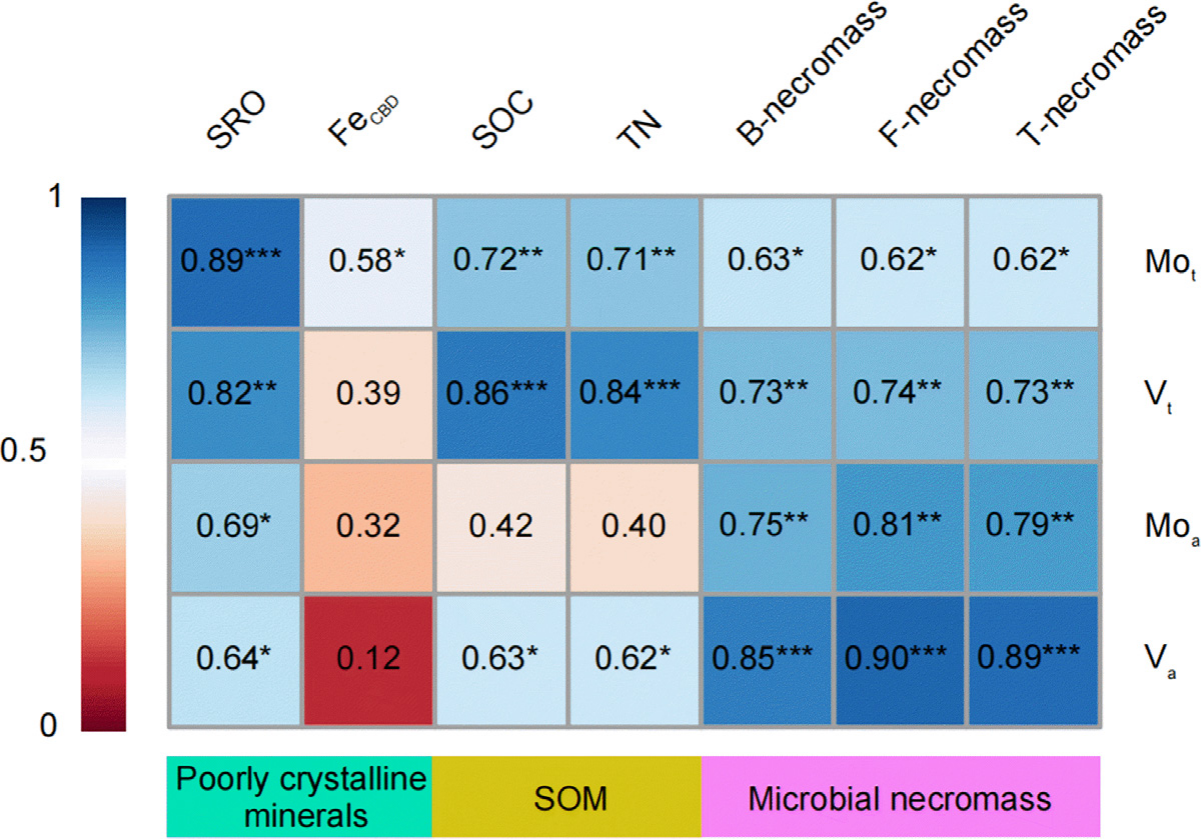
**Partial correlations between micronutrients and soil edaphic factors.** SRO (short-range-ordered) minerals are poorly crystalline Fe and Al (oxyhydr) oxides (Fe_o_, Al_o_), which are extracted using acid ammonium oxalate. Fe_CBD_ refers to the reactive Fe minerals extracted using citrate bicarbonate dithionite (CBD). SOC, soil organic carbon. TN, total nitrogen. B-necromass, bacterial necromass; F-necromass, fungal necromass; T-necromass, total necromass. A color gradient denotes Pearson’s correlation coefficients (*n* = 12). Darker shades in individual squares indicate stronger correlations between micronutrients and soil edaphic factors. *p < 0.05, **p < 0.01, ***p < 0.001. Squares without star (*) indicate insignificant relationships (*p* > 0.05).

To correlate micronutrients and their controlling factors, we further employed Partial correlation heatmap to build the relationships with various edaphic factors (Fig. 4). The SRO minerals were strongly positive correlated with the total contents and availability of Mo and V in paddy soils (Fig. 4). Micronutrients were independent on the total Fe content (Fig. S7). In contrast, Fe_CBD_ was only correlated with the Mo total content but the correlation coefficient was weaker than SRO vs Mo_t_ (Fig. 4), indicating its weak control on the micronutrients.

We showed that SOC and TN were strongly correlated with the total and bioavailable Mo and V (except for Mo_a_) (Fig. 4). Furthermore, Fe-bound OC and DOC had a stronger increase with the total and bioavailable Mo and V than SOC (Fig. S7), pointing to the key role of both stable and mobile organic compounds, rather than the bulk SOC, in the storage and bioavailability of micronutrients. Strikingly, microbial necromass closely correlated with the total and bioavailable Mo and V (Fig. 4). For the first time, we build the linkage between microbial necromass and micronutrients (i.e., Mo and V) limiting N fixation in paddy soils, suggesting a key role of microbial necromass for micronutrient retention.

### 3.5 Effects of micronutrients on nitrogen-fixing genera

**Fig. 5:**
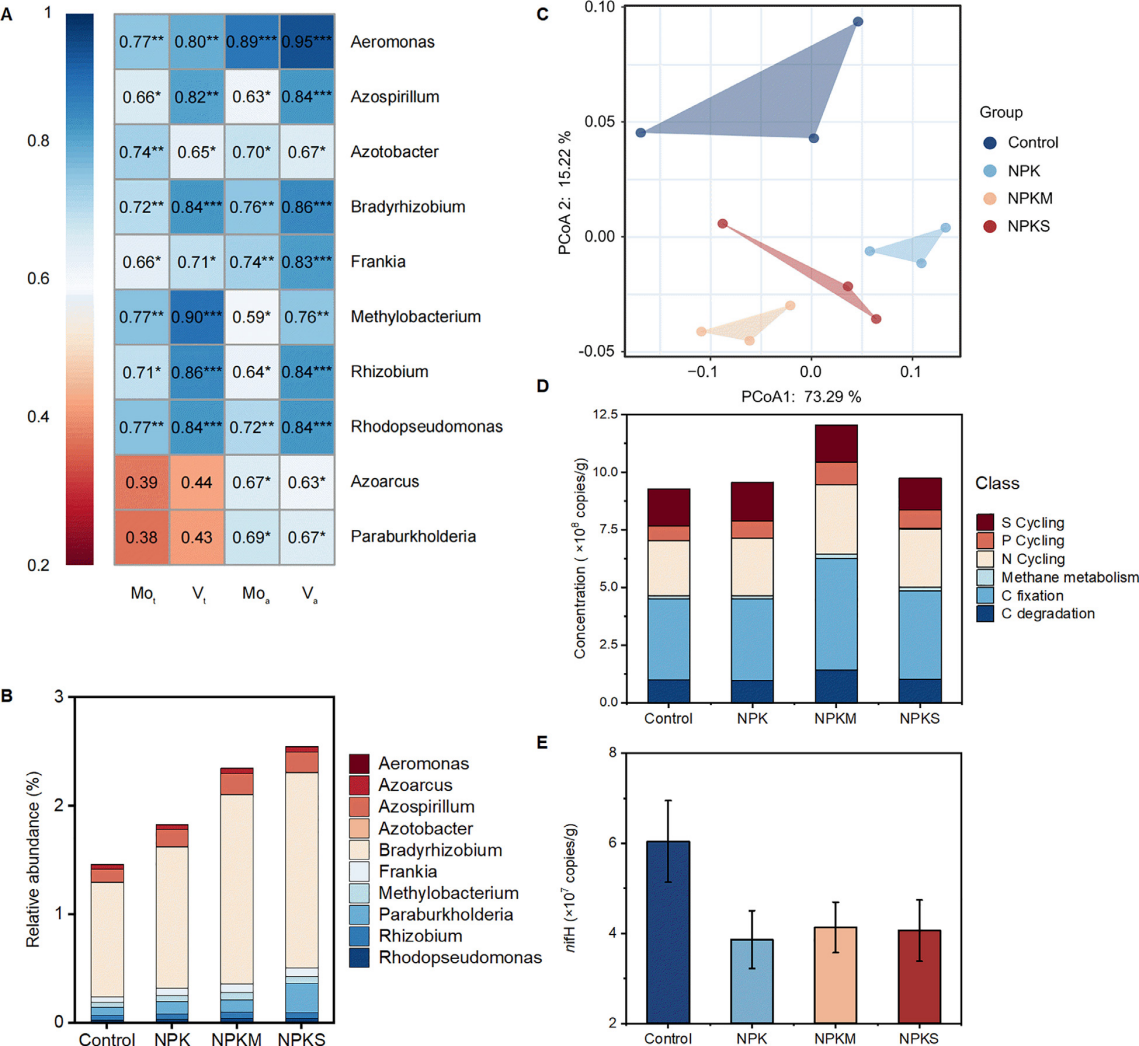
**Microbial communities and nitrogen-fixing genes in response to long-term fertilization treatments.** (A) Heatmap depicting the correlation between the 10 commonly encountered at the genus level and micronutrients (Mo and V). A color gradient denotes Pearson’s correlation coefficients (*n* = 12). Darker shades in individual squares indicate stronger correlations between micronutrients and soil edaphic factors. *p < 0.05, **p < 0.01, ***p < 0.001. Squares without star (*) indicate insignificant relationships (*p* > 0.05). (B) The top 10 commonly nitrogen-fixing genera. (C) PCoA (Principal Coordinates Analysis) plot of quantification of functional genes. (D) Stacked bar chart of the top 15 gene families. (E) Relative abundance of the N-fixing gene (*nif*H). Species abundances in A-B were obtained from metagenomic sequencing, while data in C-E were derived from HT-qPCR analysis. Control, no fertilizers; NPK, mineral fertilizers; NPKM, mineral fertilizer plus cow manure; NPKS, mineral fertilizer plus straw. Data in (E) are means ± SE (*n* = 3).

To identify which N-fixing microorganisms were affected by micronutrients, we performed metagenomic analysis and correlated micronutrients with the top 10 commonly N-fixing species at the genus level (Fig. 5A). Several N-fixation genera were strongly positive correlated with the total content and bioavailability of Mo and V, including Aeromonas, Azospirillum, Azotobacter, Bradyrhizobium, Frankia, Methylobacterium, Rhizobium, and Rhodopseudomonas (Fig. 5A). This suggests that the abundance of these above N-fixation genera was limited by the total content and bioavailability of Mo and V in paddy soils. In contrast, Azoarcus and Paraburkholderia only had a strong correlation with bioavailable Mo and V, rather than the total Mo and V (Fig. 5A). Indeed, fertilizations notably affected the abundance of functional genes (Fig. S8); as for the top 10 N-fixation genera, with the highest abundance under NPKS, followed by NPKM, NPK, and the control (Fig. 5B).

Similar to what we observed by metagenomic analysis (Fig. 5A, 5B), PCA plots, based on HT-qPCR analysis, also identified substantial difference of functional-gene structures among fertilizations, where replicates of each fertilization clustered together and soils were well separated along the second axis (Fig. 5C). Consequently, 40 years of fertilization markedly altered the abundance of functional genes in paddy soils. Analysis of the top 15 gene families indicated that NPKM had a higher abundance (12.3 × 10^8^ copies/g) of functional genes related to elemental cycling, than NPKS, NPK, and the control soil (Fig. 5D). In particular, NPKM had a slightly higher abundance of N cycling related functional genes than other three fertilizations, reflecting that long-term manure inputs could enhance their abundance, thereby promoting the N cycling process.

We further tested the effects of fertilizations on the *nif*H gene (Fig. 5E). Obviously, the average abundance of the *nif*H gene was highest in the control, and it was markedly reduced by N fertilizer inputs. However, no significant difference was observed between the four fertilizations (data not shown). As expected, the NPK had the greatest inhibitory effects on the *nif*H gene copy number. Notably, the combined application of organic and mineral fertilizers increased its abundance (Fig. 5E), suggesting that manure or straw inputs can counter one part of the inhibitory effects of NPK on N-fixing genes.

### 3.6 Direct and indirect effects of edaphic factors on nitrogen-fixing genera

**Fig. 6:**
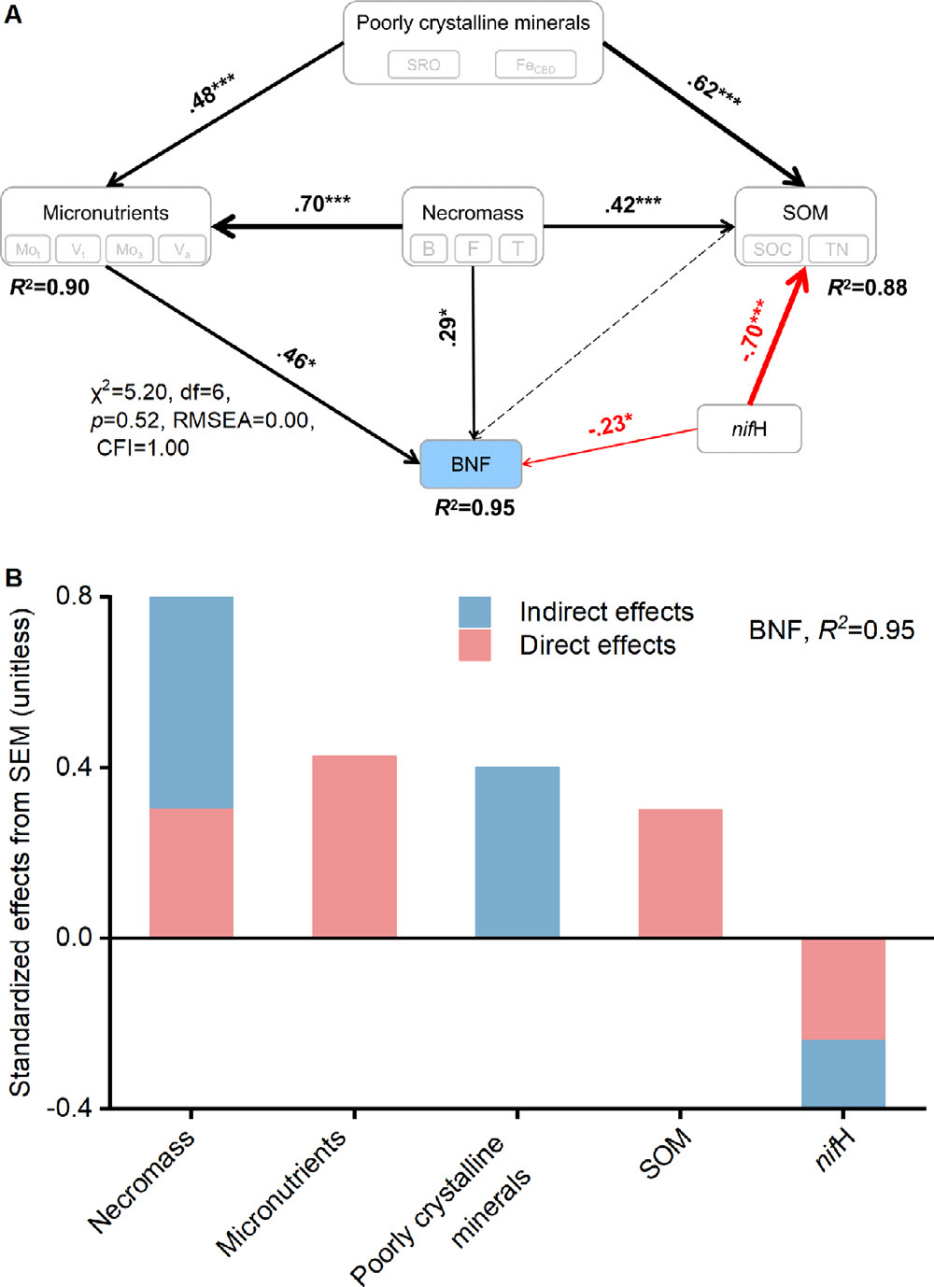
**Direct and indirect effects of edaphic factors on nitrogen-fixing genera.** (A) Structural equation model (SEM); (B) Standardized effect on N-fixing genera (Azospirillum and Bradyrhizobium). Numbers adjacent to arrows in (A) represent estimates of the standardized coefficients, analogous to partial regression weights and indicative of the effect size of the relationship. The arrow width is proportional to the strength of the coefficients. Black and red solid arrows reflect positive and negative relationships, respectively. Dashed lines indicate non-significant relationship. *p < 0.05, **p < 0.01, ***p < 0.001. The panel in (B) represents direct and indirect effects making together to total effects from variables on the abundance of nitrogen-fixing genera.

To address the effects of edaphic factors on N-fixing genera, we performed SEM analysis. Overall, the SEM explained 95.0% of the variation in N-fixing genera and provided a good fit (Fig. 6A, χ^2^ = 5.20, df = 6, *p* = 0.52, RMSEA = 0.00, GFI = 1.00). The model indicated the key role of micronutrients and microbial necromass in N-fixing genera, with the importance following the order: microbial necromass > micronutrients = poorly crystalline minerals > SOM (Fig. 6B). Unexpectedly, N-fixing genera was dominantly controlled by microbial necromass (the total standardized effect was 0.8) (Fig. 6B), highlighting an unexplored role of microbial necromass in the retention of micronutrients and thus promoting N-fixing genera in paddy soils.

Notably, microbial necromass, micronutrients, and SOM had direct positive effects on N-fixing genera (Fig. 6B). In addition, both microbial necromass and poorly crystalline minerals also indirectly affect N-fixing genera. Compared with the standardized coefficients for N-fixing genera, the indirect effects of microbial necromass (0.54) were substantially stronger than their direct effects (0.31; Fig. 6B). In contrast, *nif*H had a strong direct link with N-fixing genera via the negative association with SOC (Fig. 6A, 6B), reflecting the potential competition for micronutrients between SOM adsorption and the uptake of N-fixing genera. Collectively, SEM analysis reflected that most of the variation in the N-fixing genera, which are driven directly and indirectly by the edaphic factors through the supply or retention of micronutrients in paddy soils.

## 4 Discussion

### 4.1 Mechanisms of the retention and bioavailability of micronutrients

**Fig. 7:**
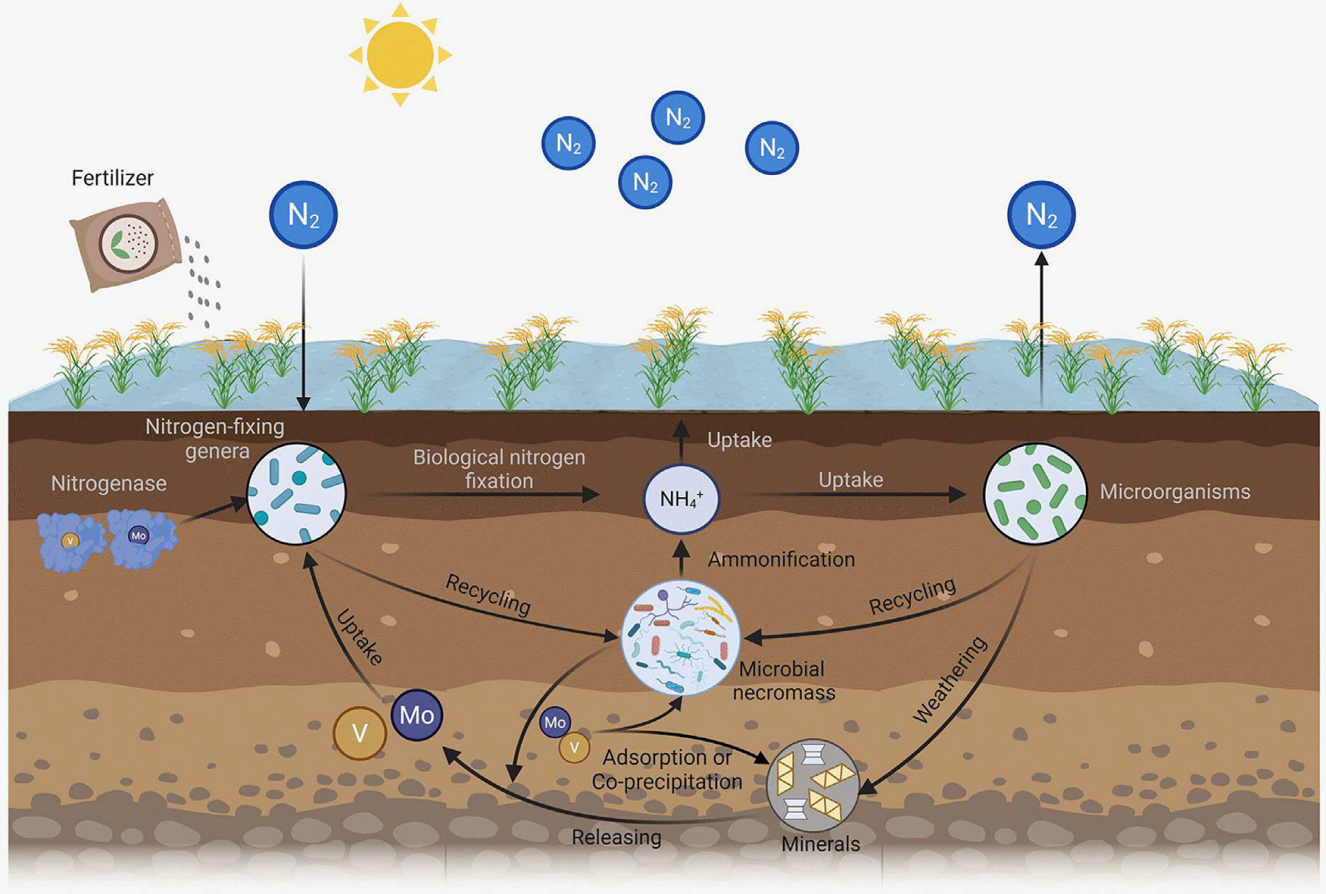
**Conceptual diagram of the retention and bioavailability of microbial necromass and minerals to micronutrients that facilitate biological nitrogen fixation in paddy soils.** In brief, following microbial death, microbial necromass emerges as an effective source for retaining and recycling micronutrients, such as Mo and V. Simultaneously, the positively charged Fe oxides, with numerous adsorption sites and the ability to form stable complexes with carbon functional groups, play a significant role in retaining micronutrients in paddy soils. Moreover, Fe minerals, particularly poorly crystalline ones, act as a metastable reservoir, supplying bioavailable micronutrients or serving as catalysts to increase the bioavailability of micronutrients in soils. These bioavailable micronutrients can be taken up by N-fixing microorganisms, contributing to the increase in biological nitrogen fixation and the abundance of N-fixing microorganisms.

In Fig. 7, we proposed conceptual framework concerning the retention and bioavailability of micronutrients in paddy soils. First, we highlight the potential dominance of microbial necromass in governing the retention of micronutrients. In soils, elements such as Mo and V predominantly exist as molybdate (MoO_4_^2−^) and vanadate (VO_3_^−^), which exhibit notable mobility and susceptibility to leaching [5]. Nevertheless, these micronutrients display a strong affinity for organic matters or tannins [12,13]. Microbial necromass, an essential component of SOC, contains numerous binding sites for organic molecules. These include positively charged glucosamine (NH_3_^+^) and negatively charged carboxyl (COO^−^) groups [37]. The NH_3_^+^ groups can directly bind with negatively charged MoO_4_^2−^ and VO_3_^−^, while the COO^−^ groups are capable of adsorbing positively charged minerals that, in turn, bind with negatively charged micronutrients. Consequently, microbial necromass plays a dual role in both the direct and indirect processes of adsorption and ligand exchange involving negatively charged micronutrients.

Given that SOC-bound Mo significantly surpasses Mo bound to Fe oxides by approximately 40 times [12], we propose that SOC largely governs the retention of Mo in paddy soils. The observed close correlation between microbial necromass and micronutrients (Fig. 4) suggests that after microbial death, microbial necromass might serve as an efficient source for recycling micronutrients [38]. It is noteworthy that fertilizer application did not alter the proportion of fungal to bacterial necromass, aligning with previous observations across global ecosystems [14]. The ratio of microbial necromass in SOC (Fig. 1B) closely mirrors findings from a global cropland meta-analysis [39]. Importantly, the lowest ratio of total microbial necromass in SOC was observed in the NPKM (Fig. 1B), akin to a global meta-analysis exploring the management effects on microbial necromass [40]. Recognizing microbial necromass as a crucial component of stable SOC that accounts for third to half of SOC [14], further investigation into the role of microbial necromass in the micronutrient retention and recycling is imperative in the coming years.

Second, Fe minerals also contribute significantly to the retention of micronutrients in paddy soils. These positively charged Fe oxides possess numerous adsorption sites and positive charges, form stable complexes directly with negatively charged C functional groups (Figs. 2 and S4) and soil micronutrients [12,13]. As ligand exchange between functional groups in SOC and hydroxyl groups in minerals stands as the principal mechanism of C stability [41], we posit that the utilization of SR-FTIR as an innovative approach is crucial for unraveling the fundamental mechanisms governing soil C stability and long-term persistence. In addition to complementing the amino sugar determinant [14,25], SR-FTIR spectromicroscopy facilitates the identification of spatial associations between key microbial components (e.g., proteins) and structural hydroxyl groups in minerals (Fig. 2). We reveal that SRO minerals exhibit a more pronounced correlation with micronutrients (Fig. 4) compared to total Fe (Fig. S7). This reflects that their higher specific surface area and binding sites [42,43] make them more prone to binding with micronutrient anions compared to other iron oxides. Our findings of decreased Fe_t_/Fe_CBD_ in soil treated with organic fertilizers (Table S4) corroborate the intensified mineral weathering [44,45], which may contribute to the formation of poorly crystalline minerals [1].

Finally, Fe minerals, particularly the poorly crystalline minerals, might serve as a metastable reservoir for supplying bioavailable micronutrients [46] or act as catalysts [19,47] to increase the bioavailability of micronutrients in soils. The high specific surface area of these poorly crystalline iron oxides [42] enables reactive SRO minerals to strongly adsorb molybdate, potentially deceasing Mo bioavailability [48]. However, our discovery of a strong positive correlation between SRO and bioavailable Mo and V (Fig. 4) contradicts this expectation. This discrepancy might be attributed to redox fluctuation cycles in Fe-enriched paddy soils, which elevate levels of reactive oxygen species (ROS) [4], subsequently raising bioavailable micronutrients. Prospective research should examine the role of ROS in modulating the bioavailability of micronutrients in paddy soils.

### 4.2 Contribution of increased micronutrients to nitrogen-fixing genera

Micronutrients (Mo and V) are taken up by N-fixing microorganisms [5] that are abundant in paddy soils. Notably, studies have indicated that the introduction of Mo into paddy soils can elevate BNF and the abundance of N-fixing microorganisms [49]. Building upon this, we propose that the heightened levels of micronutrients, including Mo and V, resulting from the application of manure or straw can foster an increase in the population of free-living N-fixing genera. This, in turn, contributes to the enhancement of BNF within paddy soils. To analyze N-fixing microbial communities in soil, the *nifH* gene stands out as an invaluable reference tool [8,50]. The direct positive influence of microbial necromass, micronutrients, and SOM on N-fixing genera is likely attributable to the provision of micronutrients, which serve as essential components of nitrogenase [8]. Additionally, the indirect impact of microbial necromass and poorly crystalline minerals on N-fixing genera likely operates through their robust capacity to retain micronutrients due to their abundant binding sites [6,13].

The most prominent suppressive effects observed in the *nifH* gene copy number due to NPK fertilization (Fig. 5) could be linked to the heightened sensitivity of nitrogenase to ammonia [51]. Consequently, excessive nitrogen fertilizer inputs lead to a decrease in both *nifH* gene abundance and the presence of N-fixing bacteria [50]. Our findings indicate that the joint application of organic and mineral fertilizers led to an increase in *nifH* gene abundance (Fig. 5E), providing an insightful avenue to curbing nitrogen fertilizer utilization by substituting a portion with manure or straw. This approach not only reduces the dependence on nitrogen fertilizers but also simultaneously boosts BNF. It is important to note that further investigation is warranted to assess parameters such as N-fixing rates or nitrogenase activity [19,52] in long-term fertilized paddy soils, potentially through methods like acetylene reduction activity assessment.

### 4.3 Significance of this study

Current food production systems are unsustainable, which are chiefly driven by the application of chemically synthesized fertilizers, especially of N [53]. This study shows that long-term manure fertilization increased the levels of micronutrients, including Mo and V, providing an effective strategy to increase trace metal retention and the abundance of N-fixing genera, thus potentially raising the BNF in paddy soils. The BNF increased with the application of manure or straw offers an unparalleled opportunity to reduce N fertilizer input while simultaneously increase crop yields. Given the fact that the amount of BNF (the average of ∼58 Tg N/year) in the terrestrial ecosystems is half of industrial N fixation globally (∼125 Tg N/year) [8], we believe that the amount of BNF in croplands can be further enlarged by agricultural practices, e.g., manure or straw amendments, based on the estimates of three billion tons of manure annually in China [54]. Besides increasing BNF in agricultural ecosystems, manure application to soils can also increase soil fertility and alleviate global food insecurity while reducing agricultural nonpoint source pollution. Overall, this study thus suggests that organic amendments to soils can be an efficient and sustainable approach to recycle micronutrients at the farm and regional scale, and to enhance BNF and crop yield and combating global food insecurity.

## 5 Conclusion

Our study experimentally demonstrated that the long-term (40 years) application of fertilizers increased the contents of microbial necromass and poorly crystalline minerals, which further contributed to the increased stable carbon burials in paddy soils. Furthermore, synchrotron radiation based spectromicroscopy analysis provided direct evidence for a ligand exchange between hydroxyl functional groups on mineral surfaces and C functional groups at the submicron scale in paddy soils. Essentially, long-term manure and straw inputs into the soil significantly increased the levels and accessibility of Mo and V. These increased levels and availability of Mo and V displayed a strong association with the presence of essential nitrogen-fixing genera, namely Azospirillum and Bradyrhizobium. Our structural equation modeling (SEM) underscored the substantial influence of microbial necromass on the abundance of nitrogen-fixing genera, revealing an underappreciated role of microbial necromass in the retention of micronutrients. This retention further promoted the growth of nitrogen-fixing genera within paddy soils. Additionally, delving into a more comprehensive comprehension of how microbial necromass turnover functions within paddy soils is of utmost importance. Such insights are crucial for refining our ability to predict the cycling of soil micronutrients in scenarios involving intermittent shifts in the soil’s redox conditions.

## Declaration of competing interest

The authors declare that they have no conflicts of interest in this work.

## Appendix Supplementary materials

Image, application 1
