# Supplementary material for: Key role of microbial necromass and iron minerals in retaining micronutrients and facilitating biological nitrogen fixation in paddy soils
Source: Fundam Res. 2024 Mar 6;5(6):2612–21. doi: 10.1016/j.fmre.2024.02.007 (PMC12744662; doi:10.1016/j.fmre.2024.02.007)
Supplement: Supplementary file 1 [file mmc1.docx]

**Supporting Information for**

**Key role of microbial necromass and iron minerals in retaining micronutrients and facilitating biological nitrogen fixation in paddy soils**

Li-Xin Xu^a^, Fei Wang^b^, Yao Yao^a^, Minjie Yao^c^, Yakov Kuzyakov^d,e^, Guang-Hui Yu^a,^*, Cong-Qiang Liu^a^

^*^Corresponding Author

Phone: +86 22 27405053; fax: +86-22 27405051; email: yuguanghui@tju.edu.cn

**Number of text pages: 19;**

**Number of Figures: 8;**

**Number of Tables: 5**

**Other Supplementary Material for this manuscript includes the following:**

Dataset S1. Relative and absolute gene abundances in paddy soils based on high-throughput qPCR analysis.

**Supporting Methods**

*Site description*

In 1983, the soil had a pH of 4.9, SOM of 21.6 g/kg, available N of 141.0 mg/kg, and available phosphorus (P) of 12.0 mg/kg. Half of the N and K fertilizers were applied as base fertilization, and the remaining fertilizers were applied during tillering, while all of the P fertilizers were applied as base fertilizer. From 1983 to 2004, a double rice-rice cropping strategy was used, but after that, a single rice crop was grown each year. The yields of straws and grains in 2022 are presented in Figure S2.

*Amino sugar determination*

Briefly, 0.5 g of dried soils were hydrolyzed at 105 °C for 8 h with 6 M HCl, and after adding an internal standard (myo-inositol), the solutions were filtered, dried at 45 °C, and re-dissolved in deionized water. The pH was adjusted to 6.6-6.8 with KOH, and the supernatant was freeze-dried. The resulting residue was dissolved in methanol and dried with N_2_ gas at 45 °C. The extracted amino sugars were derivatized using ortho-phthaldialdehyde (OPA) and separated by Hypersil GOLD C18 column (Acclaim120 C18; 4.6 mm × 150 mm, 3 µm; Thermo Fisher Scientific, Waltham, USA) at 35 °C.

*HT-qPCR analyses*

The initial enzyme activation was performed at 95 °C for 5 min, followed by 40 cycles of denaturation at 95 °C for 30 s, annealing at 58 °C for 30 s, and extension at 72 °C for 30 s. The SmartChip qPCR Software automatically generated and analyzed the melting process. Any amplification with multiple melting peaks or beyond the acceptable range of amplification efficiencies (1.8–2.2) was discarded. A detection limit of 31 threshold cycles (CT) was utilized [1].

*Soil physicochemical analyses*

Soil pH was measured using a pH electrode at a 1:2.5 soil/distilled water ratio. Total nitrogen (TN) and soil organic carbon (SOC) were determined using an element analyzer (Vario EL CUBE, Elementar, Germany). Soil dissolved organic matter (DOM) was extracted using a 1:5 w/v ratio of deionized water on a horizontal shaker (170 rpm) at 25°C for 24 h, followed by centrifugation at 3000 *g* for 10 min [2]. The resulting supernatant was filtered through a 0.45 μm polytetrafluoroethylene (PTFE) filter for dissolved organic carbon (DOC) and dissolved iron (Fe) analysis. Specifically, DOC was measured using a total organic carbon/nitrogen analyzer (1030W+1088, OI Analytical, USA). Dissolved Fe was analyzed by Inductively Coupled Plasma Optical Emission Spectrometer (5110 ICP-OES, Agilent Technologies, USA).

**Supplementary Figures**

**Fig. S1** Photo (A) of the field experiment and the designed plots (B). The field study was start in 1983 at Minhou Station, Fujian Province, China. The photo is made in October 8, 2022.

**Fig. S2** Total microbial necromass and Fe-bound OC in long-term fertilized paddy soils. T-necromass, total microbial necromass. Fe-bound OC refers to the fraction of organic carbon (OC) associated with Fe minerals and was extracted using citrate bicarbonate dithionite (CBD) method. Control, no fertilizers; NPK, mineral fertilizers; NPKM, mineral fertilizer plus cow manure; NPKS, mineral fertilizer plus straw. Significant differences between fertilization treatments were determined using one-way ANOVA followed by Duncan’s multiple range test at *p* < 0.05, in which conditions of normality and homogeneity of variance were met. Different letters above the histogram indicate statistically significant differences. Data are means ± SE (n = 3).

**Fig. S3** Dissolved iron (Fe) in long-term fertilized paddy soils. Dissolved Fe was quantified by an ICP-OES, which are extracted with deionized water. Control, no fertilizers; NPK, mineral fertilizers; NPKM, mineral fertilizer plus cow manure; NPKS, mineral fertilizer plus straw. Significant differences between fertilization treatments were determined using one-way ANOVA followed by Duncan’s multiple range test at *p* < 0.05, in which conditions of normality and homogeneity of variance were met. Different letters above the histogram indicate statistically significant differences. Data are means ± SE (n = 3).


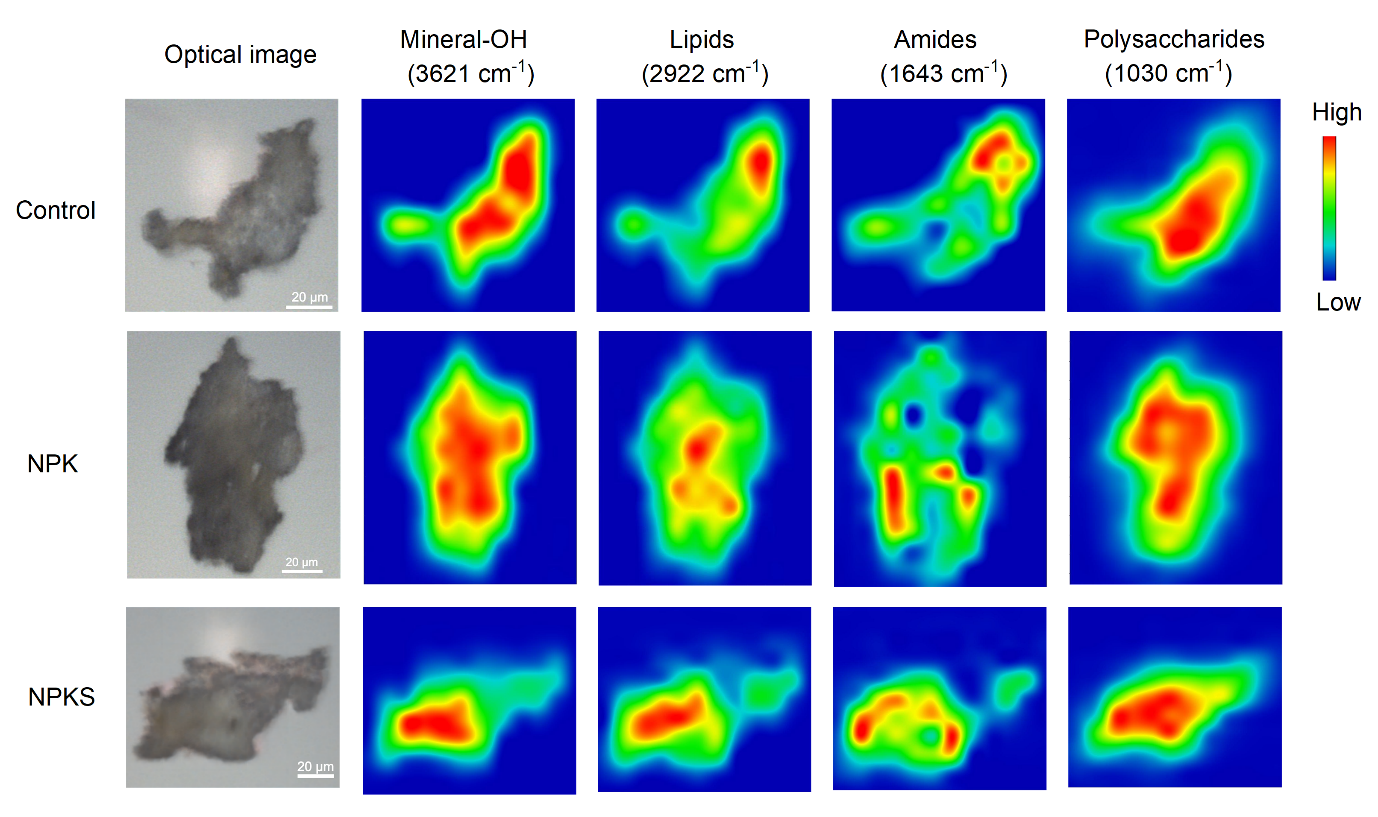


**Fig. S4** Mapping of mineral and carbon functional groups in long-term fertilized paddy soils. The colour scale is a relative scale for each peak height and does not allow quantitative comparison between peaks.

**Fig. S5** Yields of grain and straw in response to long-term (40 years) fertilization. Grain and straw were harvested in October 8, 2022. Control, no fertilizers; NPK, mineral fertilizers; NPKM, mineral fertilizer plus cow manure; NPKS, mineral fertilizer plus straw. Significant differences between fertilization treatments were determined using one-way ANOVA followed by Duncan’s multiple range test at *p* < 0.05, in which conditions of normality and homogeneity of variance were met. Different letters positioned above the histogram signify statistically significant differences, with uppercase and lowercase letters denoting straw and grain, respectively. Data are means ± SE (n = 3).

**Fig. S6** Correlation between micronutrients and grain yield. The solid lines indicate linear regressions, and the shaded areas represent 95% confidence intervals. Control, no fertilizers; NPK, mineral fertilizers; NPKM, mineral fertilizer plus cow manure; NPKS, mineral fertilizer plus straw. n = 12.


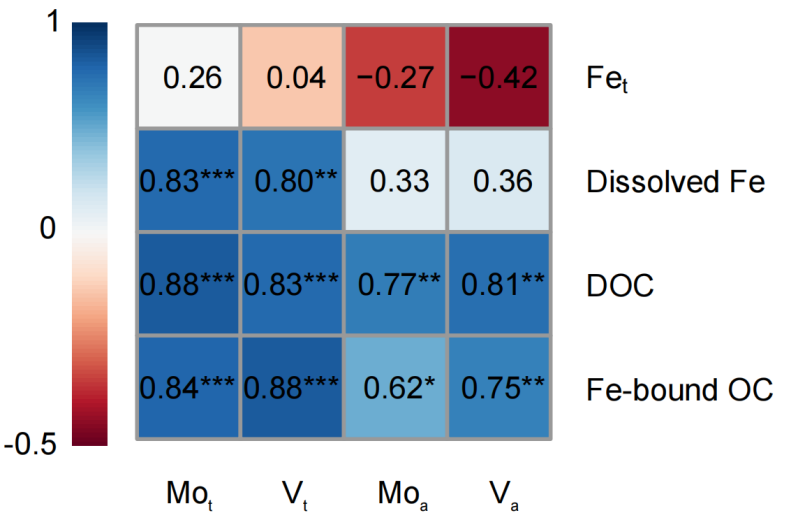


**Fig. S7** Partial correlations between micronutrients and other soil edaphic factors. These soil edaphic factors include Fe_t_, Dissolved Fe, DOC, and Fe-bound OC. Fe_t_, total Fe. DOC, dissolved organic carbon. Fe-bound OC refers to the organic carbon associated with reactive Fe minerals that are extracted using citrate bicarbonate dithionite (CBD). Mo_t_, total Mo; V_t_, total V; Mo_a_, bioavailable Mo; V_a_, bioavailable V. A color gradient denotes Pearson’s correlation coefficients (n = 12). Darker shades in individual squares indicate stronger correlations between micronutrients and soil edaphic factors. Significance is indicated as follows: **p* < 0.05, ***p* < 0.01, ****p* < 0.001. Squares without star (*) indicate insignificant relationships (*p* > 0.05).

**Fig. S8** Metagenomic analyses in response to long-term (40 years) fertilization treatments. Shannon, Simpson, PCoA (principal coordinates analysis) and Anosim analysis based on the abundance of species at the genus level. Control, no fertilizers; NPK, mineral fertilizers; NPKM, mineral fertilizer plus cow manure; NPKS, mineral fertilizer plus straw.

**Supplementary Tables**

**Table S1** Fertilization rates at the Minhou station, Fujian Province, China

| **Treatment** | **Application rates of chemical fertilizers* (kg/hm^2^)** | | | **Application rates of cattle manure or straw (kg/hm^2^)** |
| --- | --- | --- | --- | --- |
|  | **N** | **P** | **K** |  |
| Control | 0 | 0 | 0 | 0 |
| NPK | 103.5 | 11.79 | 112.5 | 0 |
| NPKM | 103.5 | 11.79 | 112.5 | 3750 |
| NPKS | 103.5 | 11.79 | 112.5 | 3660-5150 |

***** Urea, calcium superphosphate, and potassium chloride were used as the sources of N, P, and K in the field study. Control, no fertilizers; NPK, mineral fertilizers; NPKM, mineral fertilizer plus cow manure; NPKS, mineral fertilizer plus straw.

**Table S2** Nutrient contents of the applied cattle manure and straw*

| **Organic fertilizer type** | **OM** | **N** | **P** | **K** | **Total Zn** | **Total B** | **Total Cu** | **Total Fe** | **Total Mn** |
| --- | --- | --- | --- | --- | --- | --- | --- | --- | --- |
|  | **g/kg** | **g/kg** | **g/kg** | **g/kg** | **mg/kg** | **mg/kg** | **mg/kg** | **mg/kg** | **mg/kg** |
| Cattle manure | 431 | 13.2 | 3.49 | 7.42 | 98.2 | 29.6 | 18.8 | 49.3 | 381.7 |
| Straw | 651 | 7.8 | 0.92 | 22.58 | 23.2 | 3.3 | 7.6 | 56.4 | 330.0 |

* Data are collected from [3]. OM, organic matter.

**Table S3** Primer list for targeting functional genes related to nitrogen cycling

| Gene category | Gene name | Forward Primers (5'to3') | Reverse Primers (5'to3') |
| --- | --- | --- | --- |
| 16S rRNA | NA | GGGTTGCGCTCGTTGC | ATGGYTGTCGTCAGCTCGTG |
| Ammonification | ureC | AAGMTSCACGGAGGACTGGGG | AGRTGGTGGCASACCATSAGCAT |
| Anaerobic ammonium oxidation | hzo | AAGACNTGYCAYTGGGGWAAA | GACATACCCATACTKGTRTANACNGT |
| Anaerobic ammonium oxidation | hzsA | WTYGGKTATCARTATGTAG | AAABGGYGAATCATARTGGC |
| Anaerobic ammonium oxidation | hzsB | ARGGHTGGGGHAGYTGGAAG | GTYCCHACRTCATGVGTCTG |
| Assimilatory N reduction | nasA | CARCCNAAYGCNATGGG | ATNGTRTGCCAYTGRTC |
| Denitrification | narG | TAYGTSGGGCAGGARAAACTG | CGTAGAAGAAGCTGGTGCTGT |
| Denitrification | nirK1 | GGMATGGTKCCSTGGCA | GCCTCGATCAGRTTRTGGTT |
| Denitrification | nirK2 | ATGGCGCCATCATGGTNYTNCC | TCGAAGGCCTCGATNARRTTRTG |
| Denitrification | nirK3 | TGCACATCGCCAACGGNATGTWYGG | GGCGCGGAAGATGSHRTGRTCNAC |
| Denitrification | nirS1 | GTSAACGTSAAGGARACSGG | GASTTCGGRTGSGTCTTGA |
| Denitrification | nirS2 | ATCGTCAACGTCAARGARACVGG | TTCGGGTGCGTCTTSABGAASAG |
| Denitrification | nirS3 | TGGAGAACGCCGGNCARGTNTGG | GATGATGTCCACGGCNACRTANGG |
| Denitrification | nosZ1 | CGYTGTTCMTCGACAGCCAG | CGSACCTTSTTGCCSTYGCG |
| Denitrification | nosZ2 | CGCRACGGCAASAAGGTSMSSGT | CAKRTGCAKSGCRTGGCAGAA |
| Dissimilatory N reduction | napA | CTGGACIATGGGYTTIAACCA | CCTTCYTTYTCIACCCACAT |
| N fixation | nifH | AAAGGYGGWATCGGYAARTCCACCAC | TGSGCYTTGTCYTCRCGGATBGGCAT |
| Nitrification | amoA1 | STAATGGTCTGGCTTAGACG | GCGGCCATCCATCTGTATGT |
| Nitrification | amoA2 | GGGGTTTCTACTGGTGGT | CCCCTCKGSAAAGCCTTCTT |
| Nitrification | amoB | TGGTAYGACATKAWATGG | RCGSGGCARGAACATSGG |
| Nitrification | hao | TGTCATACCCGGCACAAGTTC | CATRTGGCAGAACTGRCABGT |
| Nitrification | nxrA | CAGACCGACGTGTGCGAAAG | TCCACAAGGAACGGAAGGTC |
| Organic N mineralization | gdhA | GCCATCGGYCCWTACAAGGG | ATGTCRCCNGCCGGAACGTC |

**Table S4** Soil properties after long-term (40 years) fertilization at Minhou Station, Fujian Province, China*

| **Treatments** | **pH** | **SOC** | **TN** | **C/N** | **DOC** | **Fe_t_** | **P_t_** | **Fe_t_/Fe_CBD_** |
| --- | --- | --- | --- | --- | --- | --- | --- | --- |
|  |  | **g/kg** | **g/kg** | **/** | **mg/kg** | **mg/g** | **mg/g** | **/** |
| Control | 5.73±0.02a | 10.6±0.18d | 0.85±0.02d | 12.4±0.5a | 82.3±8.15b | 20.6±0.2a | 0.13±0.01c | 3.88±0.02c |
| NPK | 5.56±0.01b | 12.8±0c | 1.11±0.02c | 11.5±0.17ab | 86.3±14.46ab | 19.3±0.12b | 0.15±0.01bc | 6.94±0.03a |
| NPKM | 5.53±0.04b | 16.4±0.04a | 1.45±0.01a | 11.3±0.07b | 128.6±17.67a | 20.4±0.16a | 0.25±0.02a | 3.59±0.03d |
| NPKS | 5.58±0.06b | 13.9±0.05b | 1.19±0b | 11.7±0.03ab | 114.6±10.4ab | 18.3±0.21c | 0.2±0.01b | 3.99±0.01b |

* Note: SOC, soil organic carbon; TN, total nitrogen; C/N, SOC/TN; DOC, dissolved organic carbon; Fe_t_, total Fe; P_t_, total P. Fe_t_/Fe_CBD_ ratios denote mineral weathering degree [4]. Control, no fertilizers; NPK, mineral fertilizers; NPKM, mineral fertilizer plus cow manure; NPKS, mineral fertilizer plus straw. Significant differences between fertilization treatments were determined using one-way ANOVA followed by Duncan’s multiple range test at *p* < 0.05, in which conditions of normality and homogeneity of variance were met. Data are shown as mean ± SE (n = 3).

**Table S5** Correlations between mineral OH and C functional groups in paddy soils as revealed by synchrotron radiation-based spectromicroscopic analysis

| **Fertilization treatments** | **Lipids vs Mineral OH** | **Amides vs Mineral OH** | **Polysaccharides vs Mineral OH** |
| --- | --- | --- | --- |
| Control | 0.91 | 0.56 | 0.87 |
| NPK | 0.96 | 0.36 | 0.93 |
| NPKS | 0.92 | 0.69 | 0.91 |

*Mineral OH, lipids, amides and polysaccharides are based on the peak height values at 3621 cm^−1^, 2922 cm^−1^ 1643 cm^−1^ and 1030 cm^−1^ in synchrotron radiation-based FTIR spectromicroscopy (SR-FTIR) images (Figure S4), respectively. N = 121, 180, and 154 for Control, NPK, and NPKS, respectively.

**References**

[1] Q.L. Chen, J. Ding, C.Y. Li, et al., Microbial functional attributes, rather than taxonomic attributes, drive top soil respiration, nitrification and denitrification processes, Sci. Tot. Environ., 734 (2020) 139479.

[2] G.H. Yu, M.J. Wu, G.R. Wei, et al., Binding of organic ligands with Al(III) in dissolved organic matter from soil: Implications for soil organic carbon storage, Environ. Sci. Technol. 46 (2012) 6102-6109.

[3] J. Yang, W. Guo, F. Wang, et al., Dynamics and influencing factors of soluble organic nitrogen in paddy soil under different long-term fertilization treatments, Soil Till. Res., 212 (2021) 105077.

[4] S. Doetterl, A.A. Berhe, C. Arnold, et al., Links among warming, carbon and microbial dynamics mediated by soil mineral weathering, Nat. Geosci., 11 (2018) 589-593.
